# Getting Your Paper Published: An Editor’s Perspective

**DOI:** 10.4103/0256-4947.75782

**Published:** 2011

**Authors:** Peter A. Hall

**Affiliations:** From the Department of Pathology and Laboratory Medicine, King Faisal Specialist Hospital and Research Center, Riyadh, Saudi Arabia

## Abstract

By means of 10 simple lessons the problems and pitfalls of getting a manuscript published are considered. Working through each lesson in turn will provide the reader with a step-by-step guide to effective publishing in the biomedical arena.

The process of scientific research is only useful if novel and robust results can be communicated to others such that the stock of human knowledge increases. Consequently publication must be seen as a central part, if not the central part, of the research process. Of course there are many benefits to authors contingent on publication, ranging from kudos to enhanced employment prospects and even tenure, but it is the communication of novel information that is paramount. Many find the process relatively easy while others struggle. Some authors are well coached by their mentors. Others have not benefited from such development, and in particular for junior researchers the key steps of grant writing and paper writing can be daunting and full of frustration. In this short review I will provide a personal perspective on the issue of writing scientific papers in the biomedical arena, based on my experiences as an author, a reviewer and an editor. I shall approach this task with a series of Ten Lessons, each of which builds into a short course that will successfully develop your writing skills

## Lesson 1: Develop your skills by reading

As with all skills the ability to write well can be learned. Some find it easy, others hard, but without doubt all can improve and develop their skills. As with all educational experiences there is a relationship between effort expended and rewards. A key ‘educational device’ in the school of scientific writing is reading! Read much and widely! Read scientific papers in front rank journals and examine closely the style and approach of authors who have succeeded. Look at the structure and clarity of language. Observe the simple structure of sentences and paragraphs. See how the well-crafted paper is concise and to the point. Notice how good authors use simple language without verbosity and flowery, overly inflated statements. There is much to be gained from time spent reading!

## Lesson 2: Have something to say

There are thousands of journals and perhaps hundreds of thousands of paper published every year. It is a sad fact that the majority of papers are not cited and it is likely that many are not read by more than a handful of people. This then begs the question as to when is something worth publishing? The key point here is ‘have something to say’! Only when you have a clear message should you begin to think about the publication process. Your message should be clear and it should be a significant addition to the literature. Consequently you need to have a good grasp of the relevant literature relating to your message, and also the techniques and methods you have used, their advantages and disadvantages, value and limitations, and the general background of the area.

## Lesson 3: Understand the structure of a scientific article

The core structure of a research article (I will not touch upon reviews here although the basic principles are common) is well established. Despite being criticized by some authorities,^1^ the basic structure was crystallized by Austin Bradford Hill^2^ in the statement:

“Why did you start, what did you do, what answer did you get and what does it mean anyway?”

This of course relates to the general paradigm of Introduction, Materials and Methods, Results and Discussion (sometimes referred to as IMRAD). The Introduction should set the scene and in a concise manner define the general background to the subject of investigation. It should not be overly long and should be in proportion to the rest of the manuscript. A useful strategy is to end this section with the question (or hypothesis) that is being posed.

The Materials and Methods of any research paper should contain enough information for the reader to understand exactly what was done. This is crucial. It may be that to save space extraneous detail might be placed in supplementary information that is only published online. This is now increasingly common but there still needs to be enough information in the paper to explain core methodologies, including patient groups. The practice of placing all Materials and Methods in supplementary material online is, in my view, unfortunate and indeed reprehensible.

The Results section should contain clear statements of all the core data and observations in a logical order. It should not contain interpretation nor materials and methods! Display items and tables should not replicate information provided in text. As with the Materials and Methods it is now common for additional data and results to be placed online and this is entirely appropriate. However, as with the Materials and Methods there should be full disclosure of the key results in the main body of the paper, again sufficient to allow the reader to understand the key data.

In the Discussion, the data should be placed in a broad context and appropriately discussed. It is common for authors to extend discussions into all sorts of tangential areas and to speculate wildly. In my view it is important to stay focused on the point of the paper. However, it should be said that it is good practice to mention and discuss potential problems and caveats of the studies reported and to consider (or at least mention) opposing or alternate views and hypotheses. A balanced paper will consider the field in the round.

A number of other elements are important in papers today. A conflict of interest (COI) statement is important. Conflicts of interest in themselves are not a bar to publication: not knowing about COI is however regrettable. A recent Commentary on this subject has been published.^3^ Statements of author contribution are increasingly seen as an important part of good writing and publication practice. Both COPE^4^ and the ICMJE^5^ have guidelines on what constitutes authorship and a statement of contribution can be useful as this forces authors to think about who has done what and who really should be an author. Clear statements about ethical approval and governance issues should be documented with relevant reference numbers. Other information that needs to be carefully collated will include the affiliations and up to date contact details of all authors as well as sources of funding and relevant acknowledgements and thanks. It is rare for research to be undertaken by one person and the many contributions of others should always be appropriately acknowledged: a ‘thank you’ goes a long way!

## Lesson 4: Understand the simple rules of writing

In Lesson 1 we considered the value of reading widely. There are general rules of writing and George Orwell outlined these.^6^ Drawing from his ideas and those of Tim Albert,^7^ one can make a number of key points.

- Never use a long word where a short one will do
- If it is possible to cut a word out, then cut it out
- Keep sentence constructions simple
- Avoid one-sentence paragraphs
- Use simple punctuation

Over and above these rules I would recommend two ‘tests’ that assist in writing. First, the ‘tell it to a friend’ test. Can you explain the points you are wishing to make in your paper in simple terms to a colleague or even to a relative who is not an expert? If you are able to do this your understanding of what you wish to convey is excellent. If you cannot then are you sure you have a full grasp of the field? Second, the ‘read it out loud’ test. Having written your paper, and once you are very happy with it, take it to a quiet place. Perhaps in an empty room or an empty field. There slowly read it out loud to yourself! You will be amazed how something you have written and looks fine on paper, sounds awful when you read it out loud. The grammatical errors and poorly contrasted sentences will jump out as you speak them!

Finally it is important to get others to read you draft manuscripts. Do not rush to send manuscripts to journals. A few days extra with input and advice from others, from mentors and colleagues can be invaluable. If nothing else they may spot typographical errors and other small points that an author can become blind to after spending days or weeks crafting their magnum opus.

## Lesson 5: How to decide where to send your paper

It is a simple fact that there exists a clear hierarchy of journals: there are those very high profile journals such as the *New England Journal of Medicine, The Lancet, Cell, Nature and Science* that command huge respect and in whose pages are often (but not invariably) carried research reports, reviews and other articles of major importance. Publishing in such high impact journals is the pinnacle of careers and inevitably few authors achieve this. There lie just below these a wide array of journals whose impact is only slightly less and also carry major impact articles. Then within any given specialty there are specialist journals and here to there is a range of ‘quality’ and impact. For example, in Pathology there are currently 71 journals listed by ISI ranging from the research journal with the highest impact: *Journal of Pathology* (Impact Factor 6.446) to the *Korean Journal of Pathology* (Impact Factor 0.064) with the mean Impact Factor of this grouping being 2.25. While Impact Factor is a widely criticized parameter^8^ it does have some utility in providing a ranking of journals. By this means potential authors can make some judgment of where their work is best placed. An important lesson it is to understand the spectrum of possible journals and the bibliometric measures that are used to create a hierarchy of them.

So then how does an author (and his colleagues) decide on where to send a manuscript? From Lesson 5 you will now have an understanding of the ‘league tables’ of journals and as a simple rule one should aim as high as possible. High impact journals will inevitably have more exposure and weight than low impact journals. But the Impact Factor is not the only variable to consider. Authors need to ask questions about the appropriateness (or fit) of their work with the journal. They need to read the aims and scope of the journal: does the proposed manuscript ‘fit’ in the journal‘s area of coverage? Authors should look at copies of the journals that they are considering and see if they publish the kinds of work that the authors are going to report. Authors need to consider some other important issues such as the time a journal takes to undertake the review process. This can be gleaned from examining published papers and examining the dates of submission and acceptance. Some journals are quick: others notoriously slow. Even when a manuscript has been accepted there is an interval between acceptance and publication: what is this for the journals you are considering? Again there is great variation. The *Journal of Pathology* currently has a mean time to first decision of 13 days. The mean time to final decision is 22 days. From acceptance to publication online of an edited but not typeset version is just a few days. Other journals are much slower. Authors would like their work disseminated as quickly as possible and so these issues will influence the decision-making process.

The final group of issues relevant to decisions regarding publication relate to costs and the nature of access. Authors should consider the quality of printing and the quality of the journal website and its online versions. Authors should consider the costs involved: are their submission charges? Are there page charges? What is the cost of color figures? Does the journal charge for supplementary material online? Over and above this authors should consider the pros and cons of open access journals, that is journals where the author pays a significant fee (typically in excess of $3000) to have the paper free to all via the Internet. All of these factors will influence the final choice of destination for your manuscript.

## Lesson 6: The instructions to authors and the need to worry about detail

Each journal will publish in hard copy and/or via its website a clear set of instructions of how a manuscript should be produced and prepared for submission to that journal. While it is the case that there are differences in detail between all journals, in reality the basic principles are the same. However it is crucial that any author reads and carefully considers all of the issues in these instructions and attends to the issues with a focus on detail. Take for example the Instructions to Authors for the Journal of Pathology (see http://onlinelibrary.wiley.com/journal/10.1002/%28ISSN%291096-9896/homepage/ForAuthors.html). Here we set the instructions out in a ‘checklist’ based manner with a range of headings that follow the normal structure of a paper. Each heading is hyperlinked to further text with explanatory detail. That detail is important and manuscripts that are submitted without attention to the various points outlined are usually returned to the authors without review. This wastes everyone‘s time! Moreover it sends a signal to the editor that the authors do not worry about detail. If they do not worry about detail in the submission process can the editor be sure they worry about detail in the research? It sends a very worrying signal!

Where do authors make the biggest mistakes? Without question the biggest errors come from (1) manuscripts, figures and tables in the wrong file format (if it says a Word file do not send a PDF), (2) incorrect font and text formats (double spaced means double spaced, not single spaced; no line numbering means no line numbering etc), (3) incorrect format of references in the text or in the bibliography, and (4) incorrect resolution for figures and other display items. With regard to the latter sadly it is the case that many authors do not realize that what looks excellent on a computer screen (resolution 72 dpi) is wholly inadequate for publication purposes, where 300 dpi are often needed. Furthermore the dimensions of an image are important. Regularly one sees figures being submitted which are 300 dpi and seemingly fine, but they are perhaps only 10 by 5 mm: when expanded to fit a full column (86mm wide) or full page (176 mm wide) the resolution falls to unacceptable levels. It seems odd that authors invest considerable time in generating data but fall at the final hurdle when preparing their figures. Again attention to detail and the meticulous following of instructions to authors is crucial to success in publication.

## Lesson 7: Understanding the steps after manuscript submission

Your manuscript has been written and after careful proofreading and worrying about all the issue in the instructions to authors, you and your co-authors are happy. Today nearly all submissions are via some online manuscript handling system. Before you begin this process make sure you have an electronic folder with all the correct files present, in the correct file format and with sensible (preferably unique) file names. Calling you main manuscript file ‘manuscript.doc’ is not smart since it is not unique. Use something in naming file that is unlikely to be confused, perhaps including the first author name, a key word, and maybe the date. Perhaps Bloggs_VEGF_hepatoma_October_2010.doc, for example. Make sure you have the final version available of each relevant file, not draft versions. Make sure track changes and comments are turned off. As with Lesson 6, worry about the detail! Most journals will want the email and relevant other contact details of all authors, so have these to hand. Again such information will be found in the instructions to authors of any reputable journal. Some journals may ask for suggestions of reviewers. If so ensure you have some sensible suggestions to make and you can sometimes suggest reviewers that you do not want to be involved: I would suggest you indicate why you have non-preferred reviewers. When suggesting reviewers avoid colleagues in your own institution or those who have obvious conflict of interest.

After successful submission all you can do is wait. The editorial team will review the manuscript and it is increasingly common for a proportion of manuscripts to be returned to authors un-reviewed (for the *Journal of Pathology* this is about 30% of manuscripts). This is usually because the manuscript is felt by the editors to be not in the scope of the journal or to in some way not be likely to have a good chance of succeeding in the review process. Hopefully this will be done in a few days and saves everyone time. Do not be dejected, but recognize the editor has probably done you a service, hastening your submission to a more appropriate journal.

After peer review, it is extraordinarily rare for a manuscript to be accepted without change: in 6 years as an Editor-in-Chief, I have only accepted two such articles! In the *Journal of Pathology* less than 25% of submitted manuscripts are ultimately accepted. A proportion of those go through one or more rounds of major revision while the rest go through one or two rounds of minor revision. Different journals have slightly different policies but for the *Journal of Pathology* minor revision usually means there is a need for some significant re-drafting or re-working of the manuscript without the need for additional experimentation. In contrast major revision usually entails the need for additional data, experiments or control studies.

After ultimate acceptance of your manuscript, it is usual for the editor to undertake some degree of editing of the text. A ‘copy-editor’, whose role is to ensure that the manuscript conforms to house style, may carry out further text editing. At that point it moves to the typesetter who creates (now by electronic means) a final version. At this point a PDF proof will be sent to the corresponding author: it is essential that they respond to the questions posed by the typesetter (usually issues of confirmation of key detail) as soon as possible. This is the last opportunity for any minor changes to be made! At some time after that the manuscript will be published online and in a print version.

## Lesson 8: Understand what editors like

Editors are simple people! They like authors to follow the instructions to authors and this is a huge step in winning over an editor. Many would be astonished by how common it is for authors to completely fail to comply with key issues in the instructions to authors (see Lesson 7). Over and above this editors like manuscripts that have a good ‘fit’ with the journal’s aims and scope and address a clear research question. A message or story that is important is desirable if not essential, and concise clear writing is important. The title should be short, informative and to the point and the abstract should be clear and comprehensive but without unnecessary material. The display items and tables should be clear and of good quality and the legends appropriate.

## Lesson 9: Be aware of what editors do not like!

The corollary of Lesson 8 is that there is a range of things that editors really do not like! Papers that do not fit with the journal, that are unoriginal, that are overly long or in any other way do not comply with the instructions to authors are the bane of the editor’s life. It may seem overly picky to some, but it is crucial to comply with formatting instructions to be found in the instructions to authors, whether they be in relation to font size, spacing, page numbers, line numbering, abbreviations, reference format and style. In addition, editors do not like manuscripts that contain poor science. For example, studies based on flawed or incorrect assumptions or that use inappropriate methodologies are problematic. In addition, poor experimental controls will compromise a potentially important study as will poor images and data presentation. Careful scrutiny of these issues by colleagues and co-authors is better than having a manuscript returned without review! Attention to detail can avoid these pitfalls (see Lesson 7).

## Lesson 10: Do not give up and understand the peer review process

Authors need to understand that most journals only accept a fraction of the submitted material. However if one is persistent and recognizes that there is a hierarchy of journals one can get your work published. A recent study of papers rejected from the *Journal of Pathology* revealed that most did get published eventually, and almost invariably in a lower impact factor journal [9]. That is to say manuscripts tend to settle at a level in relation to their perceived quality (of course authors tend to perceive their work as being world class and one has to become objective of ones efforts).

A crucial element of the publication process is the comments of reviewers and editors. The entire peer review process is intended to improve manuscripts and the quality of the scientific record. Certainly reviewers are fallible and as human beings can be influenced by external factors. However, on receipt of reviewers comments examine them with care. Even if you profoundly disagree with one or more comments, look carefully at your manuscript. Is there a possibility that the reviewer has important observations relevant to your work? Even if your paper is rejected from that particular journal you should examine your manuscript in the light of the comments. It may lead you to undertake more experiments (or make more observations). In addition at the very least it may lead you to redraft your manuscript. Use the reviewers’ comments! They are intended to help.

## Final comment

The 10 lessons in this short course will, I hope, guide authors (and especially tyros) through some of the key steps in scientific writing. But some further words might be useful. It is crucial that you read, and know, the literature that relates to your area. What you try and publish should be influenced by what others have published: be guided by that. As indicated by Lesson 1 it is important to read and read: by reading you will see good practice and hopefully develop your skills: and as with any skill practice leads to improvement, even if perfection is rarely achieved!

This review is based largely on lectures given by the author at the Workshop on Scientific Writing and the Publication Process, 23 February 2010, King Faisal Specialist Hospital & Research Center, Riyadh, Saudi Arabia.

## Conflict of interest:

*The author is Editor in Chief of the* Journal of Pathology.
